# An Efficient Deep Learning Mechanism for the Recognition of Olive Trees in Jouf Region

**DOI:** 10.1155/2022/9249530

**Published:** 2022-08-31

**Authors:** Hamoud H. Alshammari, Osama R. Shahin

**Affiliations:** ^1^Department of Information Systems, Computer and Information Sciences College, Jouf University, Sakaka, Saudi Arabia; ^2^Department of Computer Science, College of Science and Arts in Qurayyat, Jouf University, Qurayyat, Saudi Arabia

## Abstract

Olive trees grow all over the world in reasonably moderate and dry climates, making them fortunate and medicinal. Pesticides are required to improve crop quality and productivity. Olive trees have had important cultural and economic significance since the early pre-Roman era. In 2019, Al-Jouf region in a Kingdom of Saudi Arabia's north achieved global prominence by breaking a Guinness World Record for having more number of olive trees in a world. Unmanned aerial systems (UAS) were increasingly being used in aerial sensing activities. However, sensing data must be processed further before it can be used. This processing necessitates a huge amount of computational power as well as the time until transmission. Accurately measuring the biovolume of trees is an initial step in monitoring their effectiveness in olive output and health. To overcome these issues, we initially formed a large scale of olive database for deep learning technology and applications. The collection comprises 250 RGB photos captured throughout Al-Jouf, KSA. This paper employs among the greatest efficient deep learning occurrence segmentation techniques (Mask Regional-CNN) with photos from unmanned aerial vehicles (UAVs) to calculate the biovolume of single olive trees. Then, using satellite imagery, we present an actual deep learning method (SwinTU-net) for identifying and counting of olive trees. SwinTU-net is a U-net-like network that includes encoding, decoding, and skipping links. SwinTU-net's essential unit for learning locally and globally semantic features is the Swin Transformer blocks. Then, we tested the method on photos with several wavelength channels (red, greenish, blues, and infrared region) and vegetation indexes (NDVI and GNDVI). The effectiveness of RGB images is evaluated at the two spatial rulings: 3 cm/pixel and 13 cm/pixel, whereas NDVI and GNDV images have only been evaluated at 13 cm/pixel. As a result of integrating all datasets of GNDVI and NDVI, all generated mask regional-CNN-based systems performed well in segmenting tree crowns (*F*1-measure from 95.0 to 98.0 percent). Based on ground truth readings in a group of trees, a calculated biovolume was 82 percent accurate. These findings support all usage of NDVI and GNDVI spectrum indices in UAV pictures to accurately estimate the biovolume of distributed trees including olive trees.

## 1. Introduction

Unmanned aerial systems (UAS) were currently used in a number of agricultural improvement projects due to their adaptability and low-cost [1]. Typically, the unmanned aerial vehicles (UAV) will fly over the area of interest, gathering aerial photos. Flight time could variety from few minutes to many hours, regardless of the model of the UAV and also the region to be explored, amongst many other things [2]. Numerous take-offs and recoveries could well be required when employing a short-endurance UAV. The UAV operator can manually assess the sensing accuracy by transferring several low-resolution photos to a bottom either during a technological stoppage. The maximum resolution photographs are saved on the camera's storage device. When the aerial job is completed, it is transported to a desktop for postprocessing [3]. The Pix4Dmapper, Agi-soft Photo Scan correlator3D, have been used to create a mosaic in which most of the images were patched together with a substantial overlapping (60–80 percent). Despite being present, autopilot information is rarely used through this postprocessing.

**Figure 1:**
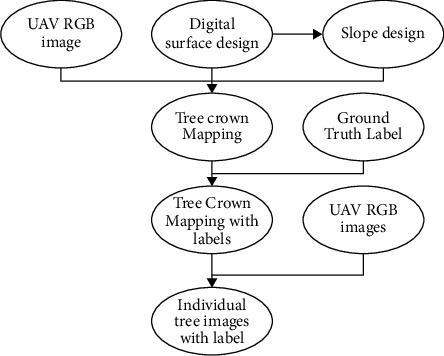
Image segmentation.

The sophistication of image processing methods [4], as well as the vast number of photographs captured in solitary aerial work (thousands), necessitates the use of sophisticated computational resources. As a result, this postprocessing should be performed on the facilities of UAV operators, employing computers having substantial quantities of storage, strong coprocessors, or computing clusters. The entire procedure could easily require 2-3 days to complete, also with annoyance that this lost time implies for the end-user. In a survey report on thermal remotely sensed related to accuracy agriculture [5], the travel time is estimated to be much longer (between 1 and 3 weeks). A geographic mosaic built using two giga-bytes of the data, for example, required 25 hours of CPU period if there is no parallel used. Furthermore, the scientists reported a lengthy (about 20 h) processing time for a series of multispectral photos (around 100) obtained using a UAV [6]. The authors created orthomosaics with ultrahigh quality using a software package running on the powerful system (a 12−core Intel *i*7 computer and 64 GB of RAM) (14.8 cm per pixel) [7].  Figure 1 summarizes the technology process of the unique tree picture segmentation and recovery approach used. First, we used a UAV image (develop at different image), a DSM, and a gradient model to separate each tree crown. Next, we created a visual representation of the ground's truth map. Finally, we labeled every tree image with such a regression coefficients label [8].

The utilization of unmanned aerial vehicle (UAV) images using near-infrared (NIR), greenish, red, and blue multispectral images is effectively implemented in personalized agriculture for evaluating plant growth and condition [9]. Spectral indicators like the normalization differential vegetative indices (NDVI) or greenish normalization differential vegetative indices (GNDVI) could be used to evaluate crop kind, productivity, and maturing phase [10]. The GNDVI indicator is added responsive to the fluctuations in a crop chlorophyll concentration which that of the NDVI indicators, but it also has a comparatively higher criterion, so it could be used in crops too closely packed canopies even in a more sophisticated growth phase, as well as to assess moisture levels and nitrogen ability to concentrate levels in plant leaf. On either hand, the NDVI value is especially useful for evaluating crop vigor during the early phases of growth [11].

Object counting has been a good computer vision issue that seeks to determine the number of things in a stationary image or a video. Object counting is indeed an established research field with numerous applications in a variety of fields, including ecological studies, population counts, microcell tallying, and vehicle counts [12]. Handcrafted characteristics (such as SIFT and HOG) were retrieved from such a stationary image to identify and measure olive trees using traditional approaches. Nevertheless, several variables including scale fluctuations, climate changes, perception abnormalities, and orientation modifications have an impact on the effectiveness of these old methods [13, 14]. Deep learning identification techniques including single shot multibox detectors (SSD) and regional-convolutional neural networks (R–CNN) have newly reached great effectiveness and proposed a possible answer to these difficulties [15]. In spite of the popularity of deep learning technologies, a standardized collection of olive trees is not accessible for deep learning purposes. As a result, we began by building a large-scale of olive database with both deep learning technology and implementations. The database is made up of 250 RGB satellite images acquired in Al-Jouf, Saudi Arabia. Satellites Pro, which offers satellite photos and mapping with most nations and towns across the world, provided the images. The olive trees were tagged with a center fact to decrease the workload and speed up the annotating process. Several olive tree deep learning applications, including identification, count, and classification, can benefit from the suggested dataset [16].

Deep learning (DL) approaches in overall, and CNNs specifically, have outperformed conventional techniques in identifying spatial characteristics from realistic RGB images. In addition, CNNs are at the cutting edge of all core computer vision applications, including classification tasks, object identification, and sample segmentation. Utilizing semantic segmentation techniques including mask regional-CNN, among the most effective CNN-based segmentation techniques, is a useful way to reliably predict olive tree crowns [17]. DL-CNNs' fundamental weakness is the systems demand a huge training of database to provide decent results. Many optimizations, such as transfer training, precise tuning, data enhancement, and possibly data fusion, were employed to solve this constraint in real systems.

The goal of this paper is to show how deep CNNs may be used to estimate the activities connected to olive-tree fields based on treetops and shadows found in ultrahigh precision photos (less than 30 cm) [18]. First, we educated CNNs to recognize olive tree crowns and shadow sections. The tree biovolumes were then estimated using the tree's crown areas, and the tree heights were calculated using the shadow distances. Earlier research on the shrubs and trees concentrated on detecting species of plants or damaging phases in photos captured by unmanned aerial vehicles (UAVs) [19]. As far as understood, it was the first study of the semantic segmentation job for plant species identification to determine tree biovolume. The following are the primary achievements of this paper.

Then, motivated by the Swin Transformer's performance, we present an actual deep learning network (SwinTU-net) for identifying and counting of olive trees. SwinTU-net is a system similar to U-net that features encoding, decoding, and skip connectors. Swin Transformer is employed instead of the convolution technique to develop good locally and globally semantic features. According to the findings of an experimental investigation on the offered database, the offered SwinTU-net approach beats the comparable research in terms of total identification, using a 0.94 percent prediction error. OTCS-dataset is a novel documented multispectral of orthoimages database for the olive tree crown identification. At two-dimensional frequencies (3 cm/pixel and 13 cm/pixel), the OTCS-dataset is divided by the semantic segmentation of mask regional-CNN model was assessed for the objectives of olive tree crown identification and shadow identification in UAV pictures. This paper presents a model that enhances identification over algorithms involving picture fusion by fusing RGB images using vegetation indices. The biovolume for olive trees was calculated using the region of the crowns and the heights deduced after their length of shadow's. The outcome reveal using NDVI and GNDVI spectrum index data with such a resolution of 13 cm/pixel is sufficient for effectively measuring the biovolume of olive plants into four subgroups of distinct spectral channels and vegetative index (RGB, NDVI, and GNDVI).

Section 2 explains previous research related to this research, and Section 3 describes the methodology of our work.  Section 4 reports on computational studies of the proposed approach, and Section 5 concludes with findings and work to be done in the future.

## 2. Related Work

An important field of research is the identifying and modeling of trees using remote sensing data for use in forest ecosystems. Earlier proposed approaches that use airborne and multispectral sensors to detect tree species with very great precision are expensive and thus inappropriate for small-scale of forest management. In this paper, we built a machine visualization scheme for the tree recognition and planning utilizing RGB images captured by the unmanned aerial vehicle (UAV) and then convolutional neural network (CNN). In this scheme, we initially determined the curve from UAV's three-dimensional framework, then instantly sectioned the UAV-RGB images such forest into a many of tree crown particles utilizing color and the three-dimensional details and the slope design, and finally applied the object-based CNN categorization to each of crown image. This scheme classified seven tree classifications, containing multiple tree species, with significantly greater than 90% of accuracy. The directed gradient-weighted category authentication modeling (guided the Grad-CAM) demonstrated that a CNN categorized trees based on their forms and leaf differences, enhancing the system's possibilities for categorizing specific trees to comparable shades in the cost-real way and significant point for forest ecosystems [20].

Coconut is one of India's most profitable crops. We establish an instinctive approach for detecting a number of the coconut trees using an UAV photo in this study. The accessibility of high spatial resolution satellite pictures enables users to create vast volumes of accurate digital imagery of vegetation regions. Today, the projected number of coconut trees may be determined in a short period using high-definition drone pictures at a minimal cost and manpower. The purpose is to develop new ways for determining coconut trees by remotely sensed. To identify coconut trees, deep learning approaches utilizing convolutional neural network (CNN) techniques are applied [21].

Cotton plant populations assessment is critical for making replanted selections within reduced plant densities locations prone to production consequences. Because measuring population of plant in a ground is a labor expensive and prone to the inaccuracy, this research proposes a fresh strategy of image-dependence on vegetation counts based on information from unmanned aircraft systems. Initially proposed image-based algorithms needed a priori knowledge of geometrical or spectral features underlying plant canopy structures, restricting the methods' adaptability in changeable field settings. In this context, a deep learning-based vegetation counting method is accessible to minimize the amount of data collected and eliminate the need for geometrical or statistics data integration. To distinguish, find, and measure cotton plants there at the seedling phase, the object recognition system You Only Look Once on version 3 (YOLOv3) and remote sensing data were used. The suggested method was evaluated using 4 distinct UAS datasets that differed in plant size, overall light, and the background luminance. The optimum crop count was found to have RMSE and R2 values of 0.50 to 0.60 plants per linear meter (numbers of plants across 1m away along with the planted row orientation) and 0.96 to 0.97, respectively. Unless there was a visible difference between cotton growing seasons, the object recognition system trained using varying plant density, ground moisture, and illumination conditions led to a decreased identification error. While cotton plants are usually separated at the germination stage, the preliminary design counting method performed strong with 0–14 individuals per linear meters of the row. This project is expected to give a computerized system for assessing plants emerging in situ utilizing UAS information [22].

Deep learning-dependence on superresolution (SR) is used in this study using Sentinel-2 photos of the Greek island of Zakynthos to determine stress levels in the supercentenarian on olive trees severe water shortages. The goal of this research to track stress levels in the supercentennial of olive trees across period and seasons. The Carotenoid Reflectance Indicator 2 (CRI 2) is derived especially using Sentinel−2 frequencies *B*2 and *B*5. CRI2 mapping with different magnifications of 10 m and 2.5 m was produced. Indeed, pictures of band *B*2 having an original pixel size of 10 m have been superdetermined to 2.5 m. In terms of spectrum *B*5 pictures, they are SR reduced between 20 m and 10 m and then to 2.5 m. Deep-learning-based SR approaches, especially DSen2 and RakSRGAN, have been used to increase pixel density to 10 m and 2.5 m, respectively. Autumn 2019, spring 2019, spring 2020, summer 2019, and summer 2020 are the five seasons evaluated. In the approach, correlations using measurement data may be used to better examine the suggested methodology's efficiency in detecting anxiety levels in extremely old olive trees [23].

The extension and development of olive farming have been related to the enormous development of Verticillium-wilt, a greatest serious fungal issue afflicting olive trees. Current research demonstrates that measures such as using new natural materials (Zeoshell ZF1) and useful microbes can reestablish vitality to afflicted trees. Nevertheless, to be effective, the above procedures necessitate the labeling of trees there in the initial stages of infestation—a work which is not only unreasonable using traditional methods (physical work), and also extremely difficult, because initial phases were impossible to detect with a human eye. The outcomes of My-Olive Grove Coach (MyOGC) scheme are discussed in this work, utilizing multispectral imagery from unmanned aerial vehicles to advance an olive meadows detection method based here on independent and automatic sorting of spectral information utilizing computer vision and the machine learning methods. A program aims to manage and evaluate the condition of olive trees, aid in forecast of Verticillium wilting development, and create decision support mechanism to assist farmers and agronomists [24].

This presents a novel deep learning system for automatic enumeration and localization of the palm trees using aerial photographs utilizing CNN throughout this research. This used two DJI UAVs to capture aerial photographs from two distinct places in Saudi Arabia for such a purpose and created a dataset of approximately 11,000 palm tree examples. Then, we used various contemporary convolutional neural network architectures to recognize palms or other forests (YOLOv3, Faster Regional-CNN, EfficientDet, and YOLOv4), and performed a comprehensive direct comparison in terms of overall correctness and inference performance [25]. YOLOv4 and EfficientDet-D5 provided the best balance of speed and accuracy (upto 99 percent mean regular precision and a 7.4 FPS). Additionally, photogrammetry principles and range adjustments were employed to dynamically infer the geographical position of observed palm palms utilizing geotagged metadata from aerial pictures. This localization approach was evaluated on two distinct kinds of the drones (DJI of Mavic-Pro and Phantom of 4-Pro) and found to have a median positioning precision of 1.6 m. This GPS tracking enables us to authenticate palm trees and estimate the number from a sequence of the drone photographs while handling image overlaps accurately. Furthermore, this novel combination of deep learning object recognition and geolocation could be used for some another entities in UAV images [26].

## 3. Methodology

### 3.1. UAV-RGB and Multispectral Images

The research area is positioned in Jouf, Saudi Arabia. The climate is the Mediterranean, with harsh summer temperatures and mild-wet winters. The median annual temperature is 400 mm, and the median yearly temperature is 15°C. Rainfed cereal agriculture and olive groves dominate the flatlands, including small areas of natural plants in the hills. To reduce interference with water supply, olive trees were spaced roughly 6 meters apart. The testing site is located within a 50-hectare olive orchard with 11,000 trees established in 2006. In this research study, we utilized a 560 m × 280 m flat rectangle comprising around 4000 trees. For the first time, remotely sensed was utilized to automatically detect trees in forestry operations. In recent times, the scientific community has prioritized tree identification and counting in crop fields. There are several approaches available for effectively recognizing and measuring olive trees. Olive tree recognition and identification from UAS photos can be divided based on image analysis approaches.

### 3.2. Proposed Model

**Figure 2:**
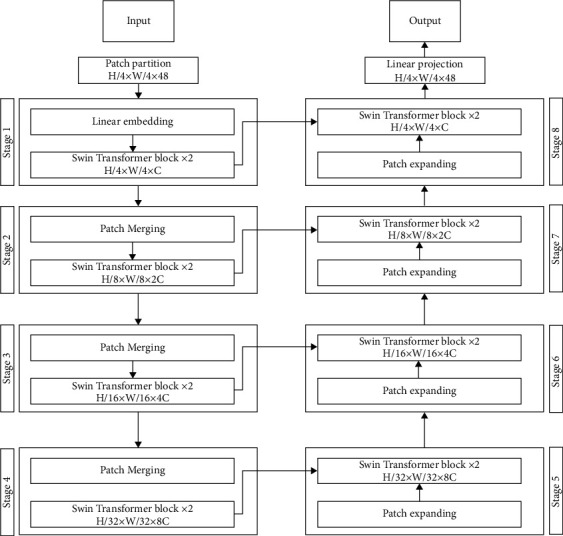
SwinTU-net model structure includes encoding, decoding, and skip connections. The Swin converter serves as the model's foundation.

The suggested SwinTU-net design, seen in Figure 2, contains encoding, decoding, and skip connectors. SwinTU-net's fundamental basis is just the Swin Transformer blocks. The encoding creates a sequence of embeddings from the inputs. The olive aerial photos have been separated into four nonoverlapping sections. Each patch already has a feature size of 4 × 4 × 3=48 as a result of this partitioning method. The predicted feature size is also turned into an unstructured length utilizing a linear-embedding layers (shown as C). Tokens (modified patched) are passed via numerous layers of Swin Converter and levels of patch combining to produce hierarchical visual features.

The patch combining layer handles downsampling and expanding size, while the Swin Transformer blocks handle feature sentence representations. This designs a synchronous transformer-based decoder influenced by the U-net. The decoding is built from Swin Transformer partitions and opposing patch widening layers [27]. The resulting perspective characteristics are combined using multiresolution characteristics output from an encoder via fully connected layers to compensate for general image loss due to downsampling. A patched increasing element, as opposed to a patched merger layer, is used particularly to promote the growth of features. The patch increasing layer resizes adjacent-dimension relevant features into large feature matrices by upsampling the frequency by two. Lastly, the last patches expansion layer is placed to the extracted features to execute four frequencies up samplings (W and H). The densities map is then created by superimposing a linear projection overlay on the front of these upsampled characteristics.

### 3.3. Swin Transformer Blocks

**Figure 3:**
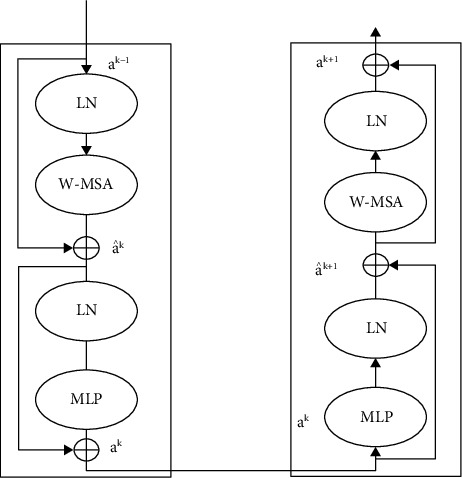
Two sequential blocks of swin transformer.

The Swin Transformer blocks, unlike typical multihead self-attention (MSA) modules, are focused on the utilization of moved panels. Every Swin Transformer block is depicted in Figure 3, which has a layer norm (LN) level, an MSA module, a residual connector, and a two of MLP levels [28]. The window-dependence on MSA (W-MSA) and shifting window-dependence on MSA (SW-MSA) components were utilized in the 2 consecutive transformers sections. Sequential Swin Transformer units could be formed utilizing a window segmentation approach as follows:

(1) $$\begin{array}{c} {\hat{a}}^{k}=W-MSA\left(LN\left(a^{k-1}\right)\right),\\ a^{k}=MLP\left(LN\left({\hat{a}}^{k}\right)\right)+{\hat{a}}^{k},+x^{k-1},\\ {\hat{a}}^{k+1}=SW-MSA\left(LN\left(a^{l}\right)\right)+a^{l},\\ a^{k+1}=MLP\left(LN\left({\hat{a}}^{k+1}\right)\right)+{\hat{a}}^{k+1}, \end{array}$$

where ${\hat{a}}^{k}$ and *a*^*l*^ are its *l*th block's outputs characteristics, (WMSA and the MLP components, correspondingly) The MSA remains determined into the similar manner as in prior studies.

(2) $$\begin{array}{c} \text{MSA}\left(E,S,V\right)=\text{softmax}\left(\frac{ES^{T}}{\sqrt{d}}+\text{Bias}\right)V, \end{array}$$

where *E*, *S*, *V* ∈ ℝ^*N*^2^×*d*^ are the inquiry, secret, and value matrix, respectively. In a window, *d* and *N*^2^ represent the inquiry or core size and the patching amount, correspondingly. The bias parameters are extracted from biases matrices $\hat{B}\in {\mathbb{R}}^{\left(2N-1\right)\times \left(2N+1\right)}$.

### 3.4. Encoding

During an encoding, two successive Swin Transformer units with such a frequency of (*H*/4) × (*W*/4), and 48 variables are performed on the incoming symbols to provide a learning algorithm. The display frequency and features dimensions remained constant. By patch combining levels, the amount of the tokens is lowered as the network grows to generate a hierarchical depiction [29]. The initial patch merging layer combines the characteristics of each set of two neighboring patches. Following that, a linear layering in 4C dimensions is added to the merging features. The outputs dimensions are set to 2C, and the token count is decreased by 2 × 2=4. Swin Transformer elements were being used to modify the characteristics while retaining the quality at H/8 W/8.

The first phase of patching combining and features modification is referred to as period 2. Because the generator is too profound to be contegrated, the operation is continued twice extra, to various output decisions of (*H*/16) × (*W*/16) and (*H*/32) × (*W*/32), in both, as “period 3” and “period 4.” The four phases would be enough to discover a deep functionality visualization because a transistor is too heavy to be cointegrated.

### 3.5. Decoding

The Swin Transformer component serves as the foundation both for encoding and decoding. In decoding, unlike encoding, the patching expanding level is employed rather than the patched combining layers to upsample the built characteristics. The patched expanding layer raises the precision of the characteristic map by rearranging nearby dimensions feature mappings and reduces the size of a characteristic by two of its input parameters. Considering the initial patches expansion layering, before upsampling, a quadratic surface is used to double the dimensions of the characteristic from ((*H*/32) × (*W*/32) × 8*C*) to ((*H*/32) × (*W*/32) × 16*C*). Use the reorganize procedure to double frequency and reduce the concept's size to a fraction of its initial dimension's((*H*/32) × (*W*/32) × 16*C*⟶(*H*/32) × (*H*/32) × 4*C*).

The skip links, such as the U-net, were utilized to feed its encoder's upsampled characteristics via the encoder's multiscale characteristics [30]. This combines the deep and shallow characteristics just to mitigate the spatial and spectral damages produced by downsampling. After such a generative model, the size of the concatenation characteristics is kept the same as the amount of the extract feature characteristics.

### 3.6. Multispectral Images and UAV RGB

This conducted two of UAV missions at 120 m altitude to collect an RGB of image with ultrahigh longitudinal precision and the multispectral imaging with an actual-high of resolution to examine the impact of deep learning methods on various spectral and spatial recommendations:

(i) A sequoia hyperspectral sensor mounted on a Parrot DiscoPro AG UAV (Al-Jouf, Saudi Arabia) that acquired four spectral features (near-infrared (NIR), red, green, and red-edge). A multispectral image had a pixel density of 13 cm/pixel [31]. The vegetative indicators described in the introduction were then calculated: the normalization differential vegetative indices (NDVI) equation (3) and the greenish normalization differences vegetative indices (GNDVI):
  (3) $$\begin{array}{c} \text{NDVI}=\frac{\text{NIR}-\text{red}}{\text{NIR}+\text{red}}, \end{array}$$
  (4) $$\begin{array}{c} \text{GNDVI}=\frac{\text{NIR}-\text{green}}{\text{NIR}+\text{green}}. \end{array}$$
(ii) This used the DJI-Phantom 4 UAV's native RGB Hasselblad 20-megapixel sensor to get higher spatial precision. The RGB image had a spatial precision of 3 cm/pixels. These RGB pictures are then transformed to the 13 cm/pixel resolution by geographical pooling to be evaluated. Granada Drone S.L. supplied the images used in both missions.

Weather parameters (sunshine and bright day) and the shooting period before nightfall are the specific criteria for current data gathering.

### 3.7. Construction of the Dataset

To create four subgroups of data to create a dataset to segment olive tree tops and tree darkness, which would allow us to examine the impact of reducing spatial resolution and acquiring spectral information. (i) RGB−3, (ii) RGB−13, (iii) NDVI−13, and (d) GNDVI−13, where 3 and 13 represent the picture pixel density in cm/pixel [32]. This produced 150 picture patches including 2400 trees for every batch of data, with 120 images (80% of a database) utilized for testing its system and 30 photos (20% of the dataset) utilized for validating the system here on olive tree crown category (Table 1) as well as olive tree shadows category (Table 2). Every image of patch comprised one to the eight of olive trees, complete using treetops and tree shadows.

**Figure 4:**
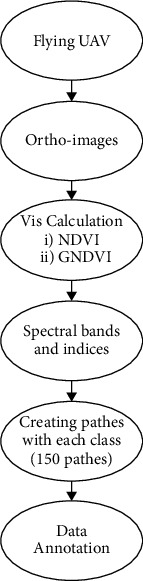
Preparing process of images.

Figure 4 depicts the overall process of creating a large dataset. Utilizing Pix4D 4.0, the initial UAV photos were integrated into an orthophoto. QGIS 2.14.21 is utilized to reduce a pixel density of an RGB−3 cm/pixel to an RGB−13 cm/pixel, as well as to calculate the NDVI and GNDI indexes. The patching was created in ENVI Classic and converted from tiff to .jpg formats (greatest appropriate arrangement for the training of deep learning techniques). The number of pixels is artificially raised to 13 cm/pixel throughout the. Tiff to .jpg transformation is done by the QGIS 2.14.21 application. VGG Image Annotator 1.0.6, standalone program for the manual feature extraction, was used to create and annotate tree crown and the tree shadow sections within every image patching. This semantic segmentation task's annotating procedure was entirely manual. In other words, the observer drew a polygon around every olive tree crown and a second-round tree shadow example. The known values developed using the VGG autocomplete feature was again saved in JSON style.

### 3.8. Mask Regional-CNN

Instance segmentation is the process of finding and separating all the pixels which make up a unique olive tree crown in UAV photos. This is among the most difficult challenges in computer vision. This employed the contemporary mask regional-CNN system (regions using convolutional neural networks) in this work that is an extension of the quicker Regional-CNN classification models [33]. Mask regional-CNN examines the contribution image and generates a three-outputs for every item class: (i) a category label indicating the object-class description, (ii) a boundary box delimiting every object class, and (iii) a mask delimiting the pixels which comprise every object class. Mask regional-CNN creates a binary mask (using values 0 and 1) for every olive tree in this study, whereas a value of 1 denotes a pixel of olive tree and value 0 denotes a pixel of nonolive tree.

To extract features, mask regional-CNN is dependent on the classification algorithm. ResNet50 CNN was used in this research to recover gradually higher-level characteristics from the weakest to deeper layer levels [34]. To enhance the categorization model's generalization performance, we evaluated the influence of data preprocessing, which contains growing the dimension of the data by implementing simple alterations including cropping (reducing columns/rows of pixel value just at edges of images), scalability, inversion, transcription, horizontal, and vertical compressive. This utilized transfer learning rather than building mask regional-CNN (inspired on ResNet50) at the start of using the database. Transfer learning involves first implementing the model's parameters using pretrained weights on the COCO-database and reskilling its system on their data source. Fine relates to retraining the last decade using a limited dataset [35].

### 3.9. Performance Evaluation of CNN

The *F*1-score measure was used to assess the effectiveness of its trained of mask regional-CNN upon this OCTS-database within the objective of an olive tree crown and shadow example of segmentation. It is calculated as the harmonics average of recall and precision. Mask regional-CNN generates three output results: a boundary area, masks, and probability in the projected class. The interaction overlap unions (IoU) or Jaccard ratio has been used to judge whether a forecast was right [36]. It is determined by the intersection of the expected and actual boundary boxes reduced by the unity. A projection is a true positive (*T*_positive_) if the IoU is greater than 50%, and a false-positive (*F*_positive_) if the IoU is minimum than 50%. The following is how IoU is measured:

(5) $$\begin{array}{c} \text{IoU}=\frac{\text{overlap area}}{\text{union area}}. \end{array}$$

A threshold quantity of 0.5 is commonly employed since it produces high indications of the score. Accuracy (equation (6)) and recall (equation (7)) are determined as follows:

(6) $$\begin{array}{c} \text{accuracy}=\frac{T_{\text{positive}}}{T_{\text{positive}}+F_{\text{positive}}}\\ =\frac{T_{\text{positive}}}{\#\text{ground\_truths}{}^{\prime }}, \end{array}$$

(7) $$\begin{array}{c} \text{recall}=\frac{{\text{T}}_{\text{positive}}}{T_{\text{positive}}+F_{\text{negative}}}\\ =\frac{T_{\text{positive}}}{\#\text{Predictions}{}^{\prime }}. \end{array}$$

Accuracy is the proportion of properly identified labels, and recall is a component of effective label retrieval. The *F*_1_ − measure is designed as the weighting factor of recall and precision (equation (8)). It considers includes *F*_negative_ and *F*_negative_ to determine a model's overall accuracy:

(8) $$\begin{array}{c} F_{1}=\frac{2\times \text{recall}\times \text{precision}}{\text{recall}+\text{precision}}. \end{array}$$

### 3.10. Execution Details

In the development, a well-known PyTorch library was employed [37]. The suggested method then is developed and validated using an NVIDIAGeForceRTX2060, GPU. Arbitrary data modifications including rotation, scale, and turning are employed before training to increase data variation. The input image is enlarged to 224 × 224, which allowed the GPU to run out of memory during training. The scores pretrained model on the ImageNet-1K is utilized to set the set of parameters [38]. During the training phase, the model is tuned for training algorithm utilizing a well SGD algorithm, using the velocity of 0.9 and weight loss of 1*e*^−4^.

## 4. Results and Discussion

This section outlines the planned olive tree datasets that will be used to test the algorithm. It also discusses the measures that were utilized to evaluate the effectiveness of the suggested model. Image segmentation findings were based on RGB and vegetative indexes. The findings of the tree biovolume computations are shown.

### 4.1. Data Collection

According to 2019 figures from the Department of Atmosphere and Waters Department in Al-Jouf, the Al-Jouf area is home to 30 million trees, the majority of that is olive trees (18 million of trees) that provide 10 thousand tons of the oil each year. As a result, collection contains 250 photos collected around Al-Jouf, KSA region, utilizing the Satellites Pro. Satellite imagery and maps are available as with most nations and towns across the world with Satellites Pro. The particular section was photographed in RGB pictures with a resolution of 512 × 512 and a bit depth of 32. To save strain and speed up annotations, the olive trees were marked using centroids. The initial step is to mark the olive photos with bounding boxes that enclose the olive trees [39]. {(*a*_*i*_, *b*_*i*_), *i*=1,2,3,4} were the four vertices of bounding boxes. In the next stage, use the given equation (9) to get the center of every box also as the center location:

(9) $$\begin{array}{c} \left(a,b\right)=\left(\frac{1}{4}\sum_{i=1}^{4}a_{i},\frac{1}{4}\sum_{i=1}^{4}b_{i}\right). \end{array}$$

### 4.2. Performance Metrics

The effectiveness of their model is determined by utilizing some performance criteria to compare different strategies on various datasets:

(i) Total accuracy (TA)
TA is the proportion of successfully predicted olive trees out of an overall amount of the olive trees. Between the designated trees, it represents the number of trees that were accurately recognized within the ground-truth dataset [40]. The respective equation is used to calculate total accuracy:
  (10) $$\begin{array}{c} \text{TA}=\frac{\text{number of estimated olive trees}}{\text{number of actual olive trees}}\times 100. \end{array}$$
(ii) Omission failure rate (OFR)
OFR is the proportion of a positive assessment individuals that were mistaken for negative test individuals. In other terms, OFR represents the proportion of times suggested algorithm flops to an identify olive trees as being. OFR is computed quantitatively utilizing the formula
  (11) $$\begin{array}{c} \text{OFR}=\frac{\text{number of omitted olive trees}}{\text{number of actual olive trees}}\times 100. \end{array}$$
(iii) Commission failure rate (CFR)
CFR is described by an occurrence of the negative specimens which were mislabeled as positive. It occurs when the output contains nonolive trees. CFR is determined mathematically utilizing the formula
  (12) $$\begin{array}{c} \text{CFR}=\frac{\text{number of false olive trees identified}}{\text{number of actual olive trees}}\times 100. \end{array}$$
(iv) Estimation failure (EF)
It corresponds to a discrepancy among the number of things detected and also a number of objects yet to be recognized. It is the ratio between the actual and predicted amount of olive trees within samples split by an actual amount of olive trees within a suggested model. The following formula is used to compute the EE mathematical model:
  (13) $$\begin{array}{c} \text{EF}=\frac{\text{number of estimated olive trees}-\text{number of actual olive trees}}{\text{number of actual olive trees}}\times 100. \end{array}$$

### 4.3. Tree Shadow and Tree Crown Segmentation Using RGB and Vegetative Index Images

Table 3 shows the effectiveness among all mask regional-CNN systems here on relevant test subgroups of its information in terms of accuracy, recall, and *F*1-measure for the tree crowns, and Table 4 shows the effectiveness of tree shadows.

As demonstrated in Table 3, several training and testing mask regional-CNN networks with tree crown segmentation had high F1 scores, exceeding 94 percent throughout all dataset groups. The F1 score was unaffected by data augmentation. The RGB subset produced the best results (F1 = 100 percent) at a spatial and temporal precision of 3 cm/pixel. In a RGB data group, increasing the display resolution from 3 to 13 cm/pixel reduced F1 by 0.42 percent without a data augmentation (plan A) and 0.86 percent with a data preprocessing (plan B). At the 13 − cm/pixel quality, the 3-band RGB images consistently outperformed the single-band NDVI or GNDVI views in terms of F1.

Nevertheless, the model was trained using data synthesis (type C, which is trained using RGB, NDVI, and GNDVI visuals simultaneously) and demonstrated similar or higher F1 than that of the trained models without data fusion (plans A and B, either with or without data preprocessing). Data aggregation boosted the F1 score by 1.76 percent for the NDVI-13 database, whereas data augmentation reduced by it 2.68 percent, as associated with training only using a NDVI-13 input data and without data preprocessing. The *F*_1_measure obtained a GNDVI database which is comparable to or higher than that obtained on the NDVI database.

As demonstrated in Table 4, all designed and evaluated mask regional-CNN models for the tree shadow identification have a high *F*_1_measure—more than 96 percent. The classifier (type D) with the greatest *F*_1_measure was designed and evaluated on the RGB visuals at 3 cm/pixel. Nevertheless, the information fusion algorithm (type *E*, which was generated on RGB, NDVI, and GNDVI images simultaneously) also demonstrated a really strong *F*_1_measure on RGB-13 cm/pixel imagery (99.58 percent). For tree shadow identification, the information fusion approach (type E) showed improvement here on RGB−13(99.58 percent) and GNDVI−13 (98.73 percent) datasets than with a NDVI−13 (96.10 percent) database.

Table 5 illustrates six olive trees that could be surveyed in the environment for open canopy area estimation combining tree circumference and tree heights fragmentation using the mask regional-CNN training set. Total precision is 94.51 percent for RGB-3, 75.61 percent for RGB-13, 82.58 percent for NDVI-13, and 77.38 percent for GNDVI-13. The system gave training and testing on the RGB images around 3 cm/pixel and had a highest total reliability for the estimating biovolume. The information fusion algorithm functioned well and achieved greater accuracy here on NDVI subgroups than with the GNDVI or RGB subgroups at 13 cm/pixel level.

### 4.4. Total Evaluation

The total prediction error for testing is 0.94 percent after implementing the proposed model. According to Table 6, for a 100% mixture of olive and nonolive trees among other items, around 0.97 percent of nonolive data is misinterpreted as an olive and 1.2 percent of the olive data were misdiagnosed as nonolive. The suggested dataset was tested, and the results indicated an overall recognition with a 0.94 percent prediction error.

An olive image, as well as ground truth and recognition findings, illustrates a combination of olive tree patterns with significant spacing among them and that those were densely established. The suggested technique appropriately defined nearly all of an olive tree, although it underestimated the number of immature and densely implanted trees.

### 4.5. Comparative Evaluation

A suggested model's findings were evaluated with those of established olive identification and enumeration algorithms. The specifications of the database, the quantity of images generated, a spectra reflecting the dimension of manufactured information, and the assessed effectiveness were all utilized in the comparisons. The outcomes of an evaluation of a proposed system to existing methodologies are shown in Table 7.

The suggested approach was validated on a large database and yielded good accuracy, as seen in Figure 4, suggesting that the methodology is efficient and robust. By reliably detecting and estimating olive trees, the suggested approach resolved weaknesses in existing approaches. This innovative algorithm for identifying and identifying olive trees was verified using RGB photos with an effective level of 98.4 percent, outperforming previous work.

**Figure 5:**
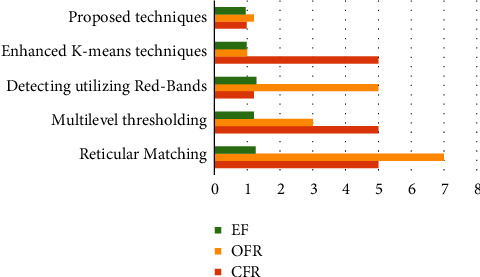
Performance evaluation.

The suggested framework exhibited the lowest total prediction error of 0.95 percent among the available strategies when tested on a huge database of olive trees as well as other surface items. It is worth mentioning that the suggested dataset contains 250 photos of olive trees as well as other things as shown in Figure 5.

There seems to be currently a plethora of low-cost RGB and the multispectral sensors which could be installed on the multirotor and secure-wing UAVs, but these images could be mechanically managed using CNN techniques for such a reason. On only side, the RGB security sensors on such a multirotor UAV may seizure considerably higher determination satellite data, which improves CNN prediction performance, but covers fewer regions (because of power limits) resulting in less costly visuals for each acre. Multispectral sensor arrays on fixed-wing drone attacks, on either side, could indeed encapsulate relatively coarse resolution satellite visuals over large regions, lowering the cost per acre while also integrating plant radiance in the near-infrared and red-edge, that also either someone to photosynthesis rate than only RGB. Merging both data sets could combine the benefits of both systems, such as increasing CNN precision, lowering cost per hectare, and including photosynthesis activities data.

The findings reveal whether CNN systems trained and tested at rather greater resolution (namely, RGB with 3 cm/pixel) achieved significantly higher accuracy (approximately 0.42 percent higher) than CNN algorithms were trained and tested at coarse grain quality (namely, RGB at 13cm/pixel). Extraspecifically, findings show which training CNN concepts on a merging among all a RGB, NDVI, and GNDVI subsamples of the images at coarse-grained pixel density (namely, 13 cm/pixel precision) outcomes in the common model with very rising accuracies (also larger than 95.0 percent and 96.0 percent for the tree crown and the tree shadow, combined) regardless of the type of image selected in the diagnostics (RGB, NDVI, or GNDVI). This extension opens the door to utilizing secure-wing multispectral or RGB images finished large regions for tree volume management at a cheaper cost per hectare, with broad consequences in accuracy agriculture, accuracy forestry, and accuracy restoration.

It is worth noting that feature extraction when applied to the mask regional-CNN model had no effect on the outcomes and even appeared to slightly lower the *F*1 score. Simulations training on the RGB image database produced a better effect across databases with such a frequency of 13 cm/pixel, indicating that the approach performs better on three-band images rather than single-band images like using both NDVI and GNDVI vegetative indexes. It can be attributed to the reality that now the supplementation data provided us with some items that resembled the plants that grow beneath and amid an olive tree, resulting in a false-positive and a fall in the total *F*1. Considering this, this concept design demonstrates how a technique of a pixel segmentation utilizing deep-CNNs may be employed efficiently in forest and agricultural situations on UAV images.

## 5. Conclusion

Finally, an effective deep learning approach (SwinTU-net) for identifying and counting the olive trees using the satellite data and UAV was developed. SwinTU-net is a system similar to U-net that features encoding, decoding, and skip links. The SwinTU-net extended a Swin Transformer block to acquire locally and globally semantic features rather than utilizing the convolution function. Furthermore, we began by generating a large-scale of olive database for a deep learning experiments and development. The fact that the CNN segmentation findings of the tree crown and the tree shadow may be utilized to estimated biovolume in numerous trees encourages additional research in this area to enhance the organization. The collection is made up of 250 RGB photos gathered in Al-Jouf, Saudi Arabia. Experimental research on the program found that the SwinTU-net model beats similar studies in terms of total identification, having a 0.95 percent prediction failure. The estimated values match the ground measures of the sample trees with a margin of error of 5.4 percent. However, there are certain disadvantages, including the difficulties in distinguishing olive trees that are near other trees. More field observations, estimates, and tests are required to have a better grasp of the possibilities of this method, which will be the subject of future research. As a result, plans include expanding the suggested dataset with more photos from diverse sources and improving the developed framework. It is proposed to evaluate trained CNN utilizing medium quality satellite data, which is of particular importance for utilizing possible outcomes over wide positions and also estimating yields and earnings for olive trees.

## Figures and Tables

**Table 1:** Patches of images and sections in four subgroups of a crown olive tree.

A subset of tree crown	Train images	Train segments	Test images	Test images	Overall images	Overall segments
RGB−3	150	500	40	130	145	650
RGB−13	150	500	40	130	145	650
NDVI−13	150	500	40	130	145	650
GNDVI−13	150	500	40	130	145	650
Overall	600	2000	160	520	580	2600

**Table 2:** Patches of images and sections in the four subgroups of an olive tree shadows.

A subset of tree crown	Train images	Train segments	Test images	Test images	Overall images	Overall segments
RGB−3	150	500	40	130	145	650
RGB−13	150	500	40	130	145	650
NDVI−13	150	500	40	130	145	650
GNDVI−13	150	500	40	130	145	650
Overall	600	2000	160	520	580	2600

**Table 3:** Mask regional-CNN segmentation effectiveness model for olive tree crown.

Testing subgroups	*T*_positive_	*F*_positive_	*F*_negative_	Accuracy	*F*_1_ measure	Recall
*(a) Models were trained on every subgroup without the use of additional data*						
Red green blue−3	130	1	0	1	1	1
Red green blue−13	120	2	3	1	0.9915	0.9959
Normalization differential vegetative indices−13	115	0	5	0.9835	0.9501	0.9661
Greenish normalization differential vegetative indices−13	120	1	12	1.00	0.9167	0.9656

*(b) Models were trained on every subgroup with the use of additional data*						
Red green blue−3	130	0	0	1	1	1
Red green blue−13	114	0	5	1	0.9835	0.9917
Normalization differential vegetative indices−13	113	15	5	0.9009	0.9835	0.9403
Greenish normalization differential vegetative indices−13	114	13	5	0.9077	0.9835	0.9538

*(c) Models were trained upon that merging of all 13 cm/pixel picture subgroups and with training data*						
Red green blue−3	120	0	0	1	0.9915	0.9957
Normalization differential vegetative indices−13	115	0	5	1	0.9667	0.9831
Greenish normalization differential vegetative indices−13	110	0	10	1	0.9084	0.9521

**Table 4:** Mask regional-CNN segmentation effectiveness model for olive tree shadow.

Testing subgroups	*T*_positive_	*F*_positive_	*F*_negative_	Accuracy	*F*_1_ measure	Recall
*(d) Models were trained on every subgroup with the use of additional data*						
Red green blue−3	130	0	0	1.0000	1.0000	1.00

*(e) Models were trained upon that merging of overall*13 cm/pixel picture subgroups and with training data						
Red green blue−3	120	0	0	1	0.9915	0.9957
Normalization differential vegetative indices−13	115	0	8	1	0.9261	0.9811
Greenish normalization differential vegetative indices−13	110	0	4	1	0.9752	0.9572

**Table 5:** Characteristics average for olive trees, where Pt represents perimeter, Ht represents height, vol represents volume, and Lt represents length.

Ground table				Types of tree crowns and tree shadows				Types of tree crowns and tree shadows				Types of tree crowns and tree shadows				Types of tree crowns and tree shadows			
				Testing using RGB−3				Testing using RGB−13				Tested using NVDI−13				Testing using GNDVI−13			
Sl. no	Pt	Ht	Vol	Pt	Lt	Ht	Vol	Pt	Lt	Ht	Vol	Pt	Lt	Ht	Vol	Pt	Lt	Ht	Vol
1	6.2	2.3	6.32	6.5	4.4	2.5	6.72	7.2	4.2	2.4	7.35	7.8	3.5	1.9	6	9.5	3.7	1.9	8.98
2	6.4	2.7	7.09	6.4	4.7	2.8	7.42	8	4.4	2.5	9.87	8.3	4.6	2.4	9.16	8.5	4.6	2.3	9.17
3	8.4	2	13.72	8.9	8.7	2.7	13.01	10	5.9	3.4	22.23	10	5.3	2.5	16.5	10.7	5.3	2.7	18.43
4	8.2	2	15.38	8.4	8.6	2.8	14.12	8.8	5.2	2.8	14.35	9.2	4.9	2.7	12.25	10.7	4.9	2.5	16.67
5	8.3	2.8	12.54	8.2	8.2	3.2	13.42	8.2	5.8	3.5	14.85	8.5	4.6	2.3	9.64	9.3	4.5	2.1	11.57
6	8.9	3	16.05	8.5	8.5	3.4	17.04	8.4	5.2	2.8	`4.75	9.3	5	2.6	13.22	10.3	5	2.5	15.94

**Table 6:** Total evaluation.

Number of images	250
Number of trees	73285
Identifies trees	72596
EF (percent)	0.95
CFG (percent)	708
OFG (percent)	913

**Table 7:** Proposed techniques comparison.

	Reticular matching	Multilevel thresholding	Detection utilizing red-bands	Enhanced *K*-means techniques	Proposed techniques
Database	QuickBird	SIGPAC viewer	SIGPAC viewer	SIGPAC viewer	UAV and satellites images pro
Spectrum	Grey-scale	Grey-scale	Red-band	RGB	RGB
Number of images	Not available	96	60	110	250

Evaluation metrics					
TA	98.0%	96.0%	Not available	98.5%	98.4%
CFR (percent)	5	5	1.3	5	0.98
OFR (percent)	7	3	5	1	1.3
EF (percent)	1.25	1.2	1.28	0.98	0.95

## Data Availability

The data used to support the findings of this study are available from the corresponding author upon request.
